# Alcohol’s Effects on the Cardiovascular System

**DOI:** 10.35946/arcr.v38.2.06

**Published:** 2017

**Authors:** Mariann R. Piano

**Affiliations:** Mariann R. Piano, Ph.D., is a Professor in and Department Head of the Department of Biobehavioral Health Science, University of Illinois at Chicago, Chicago, Illinois

**Keywords:** Alcohol consumption, alcohol use patterns, alcohol effects and consequences, cardiovascular system, heart, hypertension, coronary heart disease, stroke, peripheral arterial disease, cardiomyopathy, atherosclerosis, inflammation, alcohol-related research

## Abstract

Alcohol use has complex effects on cardiovascular (CV) health. The associations between drinking and CV diseases such as hypertension, coronary heart disease, stroke, peripheral arterial disease, and cardiomyopathy have been studied extensively and are outlined in this review. Although many behavioral, genetic, and biologic variants influence the interconnection between alcohol use and CV disease, dose and pattern of alcohol consumption seem to modulate this most. Low-to-moderate alcohol use may mitigate certain mechanisms such as risk and hemostatic factors affecting atherosclerosis and inflammation, pathophysiologic processes integral to most CV disease. But any positive aspects of drinking must be weighed against serious physiological effects, including mitochondrial dysfunction and changes in circulation, inflammatory response, oxidative stress, and programmed cell death, as well as anatomical damage to the CV system, especially the heart itself. Both the negative and positive effects of alcohol use on particular CV conditions are presented here. The review concludes by suggesting several promising avenues for future research related to alcohol use and CV disease. These include using direct biomarkers of alcohol to confirm self-report of alcohol consumption levels; studying potential mediation of various genetic, socioeconomic, and racial and ethnic factors that may affect alcohol use and CV disease; reviewing alcohol–medication interactions in cardiac patients; and examining CV effects of alcohol use in young adults and in older adults.

Data from numerous epidemiologic studies over the last two decades have revealed complex associations between alcohol use and cardiovascular (CV) conditions such as hypertension (HTN), coronary heart disease (CHD), stroke, peripheral arterial disease (PAD), and cardiomyopathy. In particular, these associations are strongly modulated by the dose and pattern of alcohol consumption. Low-to-moderate daily alcohol consumption (i.e., <15 to 20 g/day, 1 to 2 standard drinks) is associated with a reduced risk of CV disease and mortality, whereas greater amounts of alcohol consumption and a binge pattern of drinking (see definition in “Alcohol Consumption: Categories, Measurement, and Patterns”) have been linked to an increased risk. Consequently, the effects of alcohol consumption can be a double-edged sword. This article reviews these effects of alcohol consumption on CV conditions, such as HTN, CHD, stroke, PAD, and alcoholic cardiomyopathy, as well as mechanisms that may mediate the positive and the adverse effects of alcohol.

## Alcohol Consumption: Categories, Measurement, and Patterns

There are certain factors that are critically important to understanding and interpreting the data related to the health consequences of alcohol consumption. For example, how was alcohol consumption measured? What were the drink sizes and alcohol concentrations? How often did the subjects drink alcohol? What was the pattern of drinking? And was the study prospective (following subjects over time) or aggregate (pulling together data from several different studies to look for common trends)?

The way in which alcohol consumption has been measured and categorized varies, sometimes making it challenging to compare data among studies. More studies today report alcohol consumption in terms of either “drinks” or grams/units of ethanol per day or week, and alcohol consumption is measured by self-report. Most investigators also define the amount of alcohol that constitutes a “standard” drink as 12 to 15 g (with only slight variation).

Despite the progress in standardizing measurement of alcohol, studies still vary in how they define the different levels of drinking, such as low-risk or moderate and heavy drinking. Most often, low-risk or moderate drinking has been defined as 1 to 2 standard drinks per day and heavy alcohol consumption as 4 or more standard drinks per day. However, ascertaining the exact alcohol consumption threshold for determining both the benefit and risk has been challenging, and threshold levels continue to differ across studies. Additional factors make it difficult to interpret the results of these studies, including underreporting of alcohol consumption, study design characteristics (case–control studies), and unaccounted confounding variables such as socioeconomic or lifestyle characteristics that may inadvertently affect results (Emberson and Bennett 2006).

Advances are being made to address these factors. For example, alcohol consumption typically has been measured through self-report. Future studies would benefit from using direct biomarkers of alcohol consumption, such as phosphatidylethanol (PEth), to corroborate self-report of alcohol consumption and distinguish among low, moderate, and heavy alcohol consumption (Kechagias et al. 2015; Piano et al. 2015). With this in mind, the National Institute on Alcohol Abuse and Alcoholism (NIAAA) sponsored a biomarker research challenge to discover and develop biomarkers of alcohol consumption (NIAAA 2015*a*).^1^ Such a biomarker would corroborate self-reported consumption and bring more uniformity of reporting within and across studies.

Another trend in recent studies of alcohol and CV risk and disease is to include a measurement for binge drinking. In most investigations, this means consuming more than 5 standard drinks on a single occasion for men and more than 4 standard drinks for women. NIAAA defines binge drinking as a pattern of drinking alcohol that brings the blood alcohol concentration to 0.08 percent or above. A typical adult consuming the defined number of standard drinks for binge drinking would reach a blood alcohol concentration of 0.08 in about 2 hours (NIAAA 2015*b*).

## Alcohol’s Effects on Blood Pressure and Incident Hypertension

In healthy adults, consuming low-to-moderate amounts of alcohol each day typically has no short-term (i.e., acute) or substantial impact on hemodynamics or blood pressure (BP). However, data suggest that binge drinking (more than 5 standard drinks in a single sitting) is associated with transient increases in BP that range from 4 to 7 mmHg for systolic BP and 4 to 6 mmHg for diastolic BP (Potter et al. 1986; Rosito et al. 1999; Seppä and Sillanaukee 1999).

Nearly four decades ago, using data from the Kaiser Permanente Multiphasic Health Study, Klatsky and colleagues (1977) reported that HTN prevalence (BP ≥160/95 mmHg) in White and Black subjects (men and women) consuming more than 6 drinks per day was about twice as much as in nondrinkers. Using a lower systolic BP cutoff value for the diagnosis of HTN (systolic BP >140 mmHg), data from many studies generally have reaffirmed that high daily levels of alcohol consumption are associated with increased risk for HTN and overall incident HTN. However, data from current meta-analyses indicate that the risk-threshold effect or amount of daily alcohol intake associated with HTN is much lower than originally reported in the Klatsky study.

Halanych and colleagues (2010) examined the relationship between different categories of alcohol consumption and incident HTN (systolic BP ≥140 mmHg/diastolic BP ≥90 mmHg or antihypertensive medication use) among participants in the CARDIA study. The alcohol consumption categories included never-drinkers (never drank alcohol at baseline), former drinkers (no alcohol in previous year), light drinkers (<7 drinks/week for men and <4 drinks/week for women), moderate drinkers (7 to 14 drinks/week for men and 4 to 7 drinks/week for women), and at-risk drinkers (>14 drinks/week for men and >7 drinks/week for women). After adjusting for age, gender, body mass index (BMI), smoking status, family history of HTN, and numerous socioeconomic variables, alcohol use generally was not associated with 20-year incidence of HTN. The exception was in European-American women, for whom the risk of incident HTN was lower in those with any current alcohol consumption (Halanych et al. 2010). These CARDIA results differ from meta-analyses and other large prospective studies, such as the Nurses’ Health Study II (Thadhani et al. 2002) and the Physicians’ Health Study (Camargo et al. 1997; Sesso et al. 2008), which show a relationship between consuming greater levels of alcohol and incident HTN.

**Figure 1:**
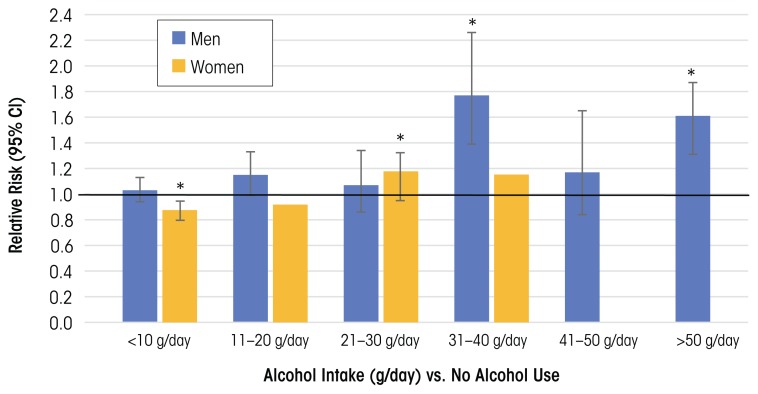
Incidence of hypertension in men and women. NOTE: * Indicates data significantly different from nondrinkers. For females, data at higher alcohol consumption levels (>40 g/day) were not analyzed. SOURCE: Data from Briasoulis et al. 2012.

In a systematic review and meta-analysis that included 16 prospective studies on the effects of alcohol consumption on the risk of HTN (systolic BP >140 mmHg/diastolic BP >90 mmHg), Briasoulis and colleagues (2012) found that consuming more than 20 g ethanol/day (~1 to 2 drinks/day) significantly increased risk of HTN in women, and higher amounts (31 to 40 g/day) increased risk of HTN in men. In women, there was a J-shaped relationship between alcohol consumption and HTN, where consumption of <10 g/day was associated with a reduced risk of HTN, whereas in men the alcohol–risk relationship was more linear (figure 1).

Results from another meta-analysis of 12 cohort studies found a similar dose–response relationship between alcohol consumption and HTN for males. A J-shaped relationship for females showed protective effects at or below consumption levels of 15 g/day (Taylor et al. 2009). These data highlight how gender may be an important modifier of the alcohol threshold level and can shape the alcohol benefit–risk relationship.

The discrepancy in findings across studies suggests that other characteristics differ among the study subjects. However (and importantly), the meta-analysis by Briasoulis and colleagues (2012) included all of the former studies and found that in the pooled analysis, for both men and women, consuming >20 g ethanol/day (~1 to 2 drinks/day) was associated with a higher risk of developing HTN.

Mori and colleagues (2015) examined the dose-dependent effects of drinking on BP measured at regular intervals in healthy premenopausal women ages 20–45. These repeated measurements allowed comparison of BP among 24 participants at 3 drinking levels, each for a 4-week consumption interval. The study included periods of low-volume and higher-volume alcohol consumption as well as of drinking alcohol-free red wine for each participant, whether or not she initially had been a lower-level or higher-level drinker as defined by the study. Awake systolic BP and diastolic BP were 2.3 mmHg/1.3 mmHg higher in women who consumed greater amounts of alcohol (146 to 218 g/week, ~2 to 3 standard drinks/day) than in those who drank less (42 to 73 g/week, ~0.5 to 1 standard drink/day) or none at all. There was no BP-lowering effect with lower alcohol amounts. In women, these findings support the data from meta-analyses and prospective studies, suggesting that greater amounts of alcohol consumption may increase BP and contribute to the development of HTN. However, findings from this study do not support a BP-lowering effect at the lower level of alcohol consumption (42 to 73 g alcohol/week, or ~3 to 5 standard drinks). Interestingly, in the Mori study, higher alcohol consumption was associated with a 10 percent increase in high-density lipoproteins (HDLs, which remove cholesterol from the blood and are associated with reduced risk of atherosclerosis and heart disease) and a 14 percent reduction in levels of fibrinogen (a glycoprotein that helps form blood clots).

Most of the studies included in the Briasoulis meta-analysis examined the effects of alcohol consumption on subjects with Stage I HTN (BP >140/90 mmHg). Fan and colleagues (2014) examined the prevalence of prehypertension (systolic BP/diastolic BP 120 to 139 mmHg/80 to 89 mmHg) and found 52 percent of male current drinkers and29 percent of female current drinkers had pre-HTN.

To summarize, in both men and women, alcohol consumption at levels above about 1 to 2 drinks per day is associated with HTN. The alcohol–risk relationship tends to be J shaped in women and linear in men. More research is needed to determine if certain ethnic or socioeconomic groups are more vulnerable to alcohol-induced HTN. The American Society of Hypertension and the International Society of Hypertension recommended that men limit their alcohol consumption to no more than 2 drinks a day, and women to no more than 1 drink a day (Weber et al. 2014). To put the importance of BP control into perspective, at a population level, a 2-mmHg increase in BP increases mortality from stroke by 10 percent and from coronary artery disease (CAD) by 7 percent (Lewington et al. 2002; Mori et al. 2015).

## Potential Biologic MechanismsUnderlying Alcohol-Induced BP Effects

Several mechanisms may underlie alcohol’s effects on blood pressure. These include impairments in cells that lead to buildup of plaque in arteries (i.e., through alterations in endothelial cell function and nitric oxide availability), and disruptions in arterial-vascular function (i.e., through myogenic mechanisms and changes in baroreceptor function), and hormonal imbalances that control the body’s fluid and BP regulation (through the renin–angiotensin–aldosterone system [RAAS]). Some adverse BP-related mechanisms that may be triggered by alcohol include changes in intracellular calcium levels, baroreflex control, and heart rate and activation of other neurohormonal systems besides the RAAS, such as the sympathetic nervous system (Marchi et al. 2014).

Several reports indicate that alcohol first exerts a seemingly positive effect, followed by a more negative impact (i.e., it is biphasic) on the endothelial–nitric oxide–generating system. The endothelium is a key regulator of vascular function. Endothelial dysfunction is an early indicator of blood vessel damage and atherosclerosis, as well as a strong prognostic factor for future CV events (Deanfield et al. 2007; Ras et al. 2013). Low-to-moderate levels of alcohol consumption may initially improve endothelial function, whereas high daily levels and binge drinking may impair it.

Other studies have shown that low-to-moderate concentrations of ethanol (20 mM) increase endogenous nitric oxide synthase (eNOS) expression in certain cells (i.e., human umbilical-vein endothelial cells) (Liu et al. 2002). Low-to-moderate ethanol consumption in rats (36 percent of caloric intake) for 6weeks increased nitric oxide production and eNOS expression in the aortic vascular wall (Kleinhenz et al. 2008). Nitric oxide helps regulate vascular tone. eNOS has a protective function in the cardiovascular system, which is attributed to nitric oxide production. However, higher daily ethanol (blood alcohol levels >29 mM) for 6 weeks in another animal model was associated with decreased eNOS expression, increased release of endothelial-derived vasoconstrictor prostanoids, and greater responsiveness of mesenteric arterioles to phenylephrine (Tirapelli et al. 2008). Taken together, these findings show lower amounts of alcohol may have a positive effect on nitric oxide signaling, but higher amounts alter this system and change arteriolar reactivity, which may led to an increased risk for HTN.

In humans, endothelial function is assessed by measuring the widening (i.e., dilation) of the brachial artery under different conditions. Some research noted that endothelial function is impaired in abstinent individuals with a long-term history of alcohol abuse or alcoholism(Di Gennaro et al. 2007, 2012; Maiorano et al. 1999). Other studies have examined the effect of a single binge-drinking episode and found impairment in brachial artery endothelial-dependent and -independent vasodilation (Bau et al. 2005; Hashimoto et al. 2001; Hijmering et al. 2007). Therefore, as in animal studies, the effects of ethanol on endothelial function in humans likely depend on the dose and duration of ethanol consumption.

Vascular wall oxidative stress also is a key mechanism in ethanol-induced HTN. Oxidative stress is an imbalance between production of free radicals and the body’s ability to detoxify or fight off their harmful effects through neutralization by antioxidants. Various studies with animals and humans indicate that ethanol can increase the development of reactive oxygen species (ROS), leading to increases in redox-signaling pathways and decreases in protective antioxidant levels. Alcohol also can increase levels of co-enzymes or reducing equivalents (e.g., reduced nicotinamide adenine dinucleotide phosphate [NADPH]), which lead to increases in ROS formation and decreases in eNOS activity (Ceron et al. 2014). Several excellent reviews offer more detailed assessments of vascular cellular mechanisms (Cahill and Redmond 2012; Husain et al. 2014; Marchi et al. 2014; Toda and Ayajiki 2010).

## Alcohol, CHD, and Stroke

The relationship between and among alcohol consumption, CHD, and stroke has been extensively investigated. Many of these studies have been conducted in middle-aged and older people and across populations. These studies have used ecologic designs (in which at least one risk-modifying health factor is measured at the group level); case–control designs (which compare clinical cases with control subjects to determine if an exposure is associated with an outcome); and longitudinal research designs (which gather data from the same subjects repeatedly over a length of time). Examples include the Health Professionals Follow-up Study (Harvard School of Public Health 2016*a*), the Nurses’ Health Study (Harvard School of Public Health 2016*b*), the Framingham Heart Study (National Heart, Lung, and Blood Institute and Boston University 2016), the British Doctors Study, the Physicians’ Health Study,^2^ the Copenhagen City Heart Study (Schnohr et al. 2002), and INTERHEART (Leong et al. 2014). The availability of these diverse datasets has allowed for completion of several comprehensive systematic reviews and meta-analyses of alcohol, CHD, and stroke relationships. This section summarizes data from meta-analyses, along with data from large international studies such as INTERHEART (Leong et al. 2014) and other recent studies using new methodologies such as Mendelian randomization (reviewed below in “Alcohol Consumption and Total Stroke Incidence and Prevalence”).

## Alcohol Consumption and CHD

**Figure 2:**
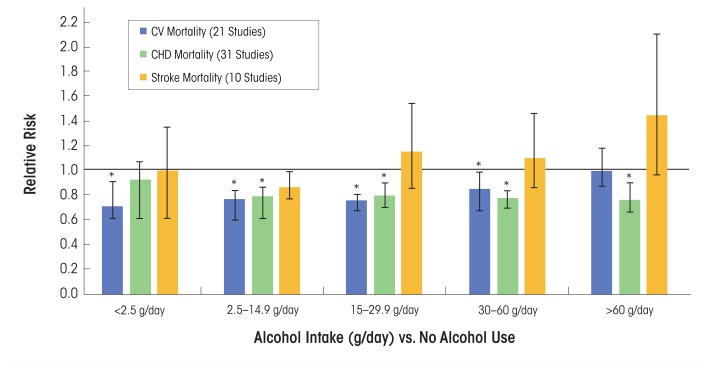
Relative risks (95% confidence intervals) for cardiovascular (CV), coronary heart disease (CHD), and stroke outcomes. SOURCE: Data used from Ronksley et al. 2011.

In a comprehensive systemic review and meta-analysis, Ronksley and colleagues (2011) analyzed data from several prospective studies (*n* = 84), of which 40 percent reported on all-male cohorts, 7 percent reported on women only, and 53 percent included men and women. The most data-adjusted analysis, which included both men and women, noted that various alcohol consumption levels (g/day) among active drinkers compared with nondrinkers were associated with a reduced relative risk for CV mortality, incident CHD, and CHD mortality. Alcohol consumption levels between 2.5 g/day and 30 to 60 g/day (<1 standard drink/day to ~5 drinks/day) were cardioprotective for both CV mortality and CHD mortality (figure 2). However, the association between alcohol consumption and CV mortality was insignificant when alcohol consumption was >60 g/day, but remained significantly reduced for CHD mortality.

Findings from INTERHEART, a 52-country case–control study of individuals with first myocardial infarction (MI), also supported the fact that “alcohol use” was associated with a reduction in the odds ratio for first-time MI (table 1) (Leong et al. 2014). In addition, in the 24 hours after alcohol use, there was no effect of “alcohol use” on risk of MI.

It is important to note that, unlike other studies with more discrete alcohol consumption categories, alcohol use was nonspecifically defined in INTERHEART as the consumption of at least 1 alcoholic beverage within the previous 12 months (Leong et al. 2014). Interestingly, the strength of this association was not consistent across different geographic regions. Alcohol use was protective against CHD for subjects in most countries, except for people of South Asian ethnicity living in South Asia (India, Bangladesh, Nepal, Pakistan, and Sri Lanka). INTERHEART results also suggested that the protective effect of any alcohol use against MI was greater in women and those over age 45. Finally, data from INTERHEART support the finding that the risk of MI is increased in the 24 hours after consumption of 6 or more drinks, suggesting that binge drinking increases MI risk (table 1).

Mostofsky and colleagues (2016) conducted a systematic review and meta-analysis (*n* = 23 studies) to examine the effects of alcohol consumed in the 24 hours before MI onset. These investigators found a U-shaped relationship between alcohol intake and MI risk, with the greatest benefit occurring after ~28 g of alcohol (~2 drinks, or moderate consumption) in 1 day and a higher risk after ~108 g (~9 drinks, or heavy consumption) in 1 day. Within a week after alcohol consumption, there was a lower risk of MI with moderate alcohol consumption but a greater risk with heavy alcohol consumption (Mostofsky et al. 2016).

## Alcohol Consumption and Total Stroke Incidence and Prevalence

Many epidemiologic studies also have been conducted to evaluate the association between alcohol consumption and total stroke incidence and prevalence, as well as the separate effects on specific stroke subtypes (e.g., ischemic and hemorrhagic). In the same systematic review and meta-analysis noted above, Ronksley and colleagues (2011) systematically examined the relationships between and among different levels of alcohol consumption and incident stroke and stroke mortality. They found a decrease or no effect on relative risk for incident stroke and stroke mortality, respectively, at <2.5 g and 2.5 to 14.9 g of alcohol/day, and almost no overall associations of alcohol consumption with levels between 15 to 29.9 g and 30 to 60 g of alcohol/day (figure 2). For heavier drinkers (60 g/day) the risk for incident stroke was greater compared with abstainers, and the risk for stroke mortality was about one and a half times greater (figure 2). A subanalysis of stroke subtypes revealed that when pooling the risk among current alcohol drinkers compared with nondrinkers, the risk was actually higher for incident hemorrhagic stroke than for ischemic stroke (*n* = 12 studies) (Ronksley et al. 2011).

In another analysis of these same studies, Zhang and colleagues (2014) found a J-shaped relationship between alcohol intake and stroke. Compared with no alcohol intake, consumption of 0 to 20 g/day (or <2 drinks) was associated with a reduced risk of total stroke, ischemic and hemorrhagic stroke, and stroke mortality. However, alcohol levels >30 g/day (>2 drinks), and in particular >45 g/day (>3 drinks), were associated with increased risk of all stroke outcomes. It is important to note that in this meta-analysis, “low-alcohol intake” also included “no alcohol intake” or 0 g/day (Zhang et al. 2014).

## New Methods for Analyzing Alcohol Consumption and Stroke-Related Outcomes

Investigators are using new methods to examine the relationship between alcohol consumption and CV outcomes. One such method includes Mendelian randomization, an epidemiologic study design that incorporates genetic information into traditional epidemiologic methods. Mendelian randomization offers an opportunity to test the relationship between a causal factor (e.g., alcohol consumption) and a specific outcome (e.g., CV disease). Holmes and colleagues (2014) used Mendelian randomization to determine if a relationship exists between drinkers with a certain variant in the alcohol dehydrogenase *ADH1B* gene (rs1229984), which is associated with reduced alcohol consumption, and the likelihood of having fatal or nonfatal CHD and stroke. The investigators found that individuals with the A allele variant *ADH1B*rs1229984 consumed less alcohol and had a reduced risk of CHD and ischemic stroke compared with noncarriers. This implies that lower alcohol consumption, even in light-to-moderate drinkers, was beneficial for CV health. These results challenge the findings from several of the studies mentioned earlier that support a cardioprotective effect of low-to-moderate alcohol consumption. They also suggest that traditional epidemiologic studies may not capture important nuances related to selection bias or other errors that can affect study results (Chikritzhs et al. 2015; Holmes et al. 2014).

## Conclusions About Alcohol Consumption, CHD, and Stroke

Based on these findings in both men and women, alcohol consumption of about 1 to 2 drinks per day is associated with a decrease in CHD. However, alcohol consumption may not have cardioprotective effects in certain racial or ethnic groups, such as in people of South Asian ethnicity living in South Asia (Leong et al. 2014).

Although results related to levels of alcohol consumption and stroke events are less clear, some conclusions can be drawn. Approximately 1 to 2 drinks per day may have no effect on or lead to a slight reduction in stroke events; however, greater daily alcohol levels increase the risk for all stroke events and incident stroke types. In terms of stroke subtypes, compared with nondrinkers, current alcohol drinkers have an increased risk (~14 percent) for hemorrhagic stroke (Ronksley et al. 2011).

## Alcohol and Heart Failure

Several studies and meta-analyses have been conducted to determine the relationship between alcohol consumption and the risk of developing heart failure in healthy subjects, as well as in those with a history of MI or CHD. Heart failure is a syndrome that often results from an MI or CHD. Studies also have examined the “safety” of alcoholic beverage consumption in subjects with heart failure.

In a meta-analysis of prospective studies (*n* = 8) of healthy people ages 21–81, Larsson and colleagues (2015) reported that, compared with nondrinkers, the risk for heart failure across different levels of alcohol consumption was greatest for those consuming 12 drinks per week, intermediate for those consuming 3 drinks/week as well as for those consuming 14 drinks/week, and least for those consuming 7 drinks/week. Based on dose–response analysis, consumption of 7 drinks/week was associated with a 17 percent lower risk of developing heart failure.

In contrast, Wannamethee and colleagues (2015) recently examined different levels of alcohol consumption and risk for heart failure in an older population (mean age ~68) and found no evidence that light-to-moderate drinking had a protective effect on incident heart failure in this age group. On the other hand, drinking ≥5 drinks/day (or ≥35 drinks/week) was associated with a significant risk of heart failure. In subjects with reduced ejection fraction–related heart failure (with the fraction of outbound blood pumped from the heart with each heartbeat, or ejection fraction, at <35 percent) and a history of ischemic heart disease or CAD (mean age 59), Cooper and colleagues (2000) found that light-to-moderate drinking (1 to 14 drinks/week) was associated with a significant reduction in progressive heart failure and hospitalization. However, there were no positive effects in subjects with mechanical or electrical dysfunction of heart muscle, or nonischemic heart disease, and although not significant, there was a slight risk for hospitalization for heart failure.

More recently, Cosmi and colleagues (2015) examined the effects of daily wine consumption in subjects enrolled in an Italian trial of heart failure patients (mean age ~67), most of whom had reduced ejection-fraction heart failure. Different levels of daily wine consumption (i.e., sometimes, 1 to 2 glasses/day, and ≥3 glasses/day) had no effect on fatal or nonfatal outcomes (e.g., hospitalization for a CV event). Subjects who drank wine more often, however, were less likely to have symptoms of depression and more likely to have a better perception of health status. They also had lower levels of circulating inflammatory markers, such as C-terminal proendothelin-1 and pentraxin-3 (Cosmi et al. 2015).

Thus, low levels of alcohol consumption (1 to 2 drinks, but not every day) in patients with heart failure may not exacerbate the condition, especially in those with heart failure attributable to ischemic CHD. Because heart failure patients usually are older (over age 65) and often are prescribed numerous medications, both the effects of age and of medication use should be carefully considered by patients, clinicians, and researchers.

## Alcohol and PAD

Compared with CHD and stroke, the relationship between alcohol consumption and PAD has been examined less often, and to date there are no meta-analyses or systematic reviews. PAD is used broadly to refer to pathophysiologic conditions affecting the arterial system. Fifteen years ago, two large prospective studies, one from the United States (the Strong Heart Study) (Fabsitz et al. 1999) and one from Europe (the Rotterdam Study) (Vliegenthart et al. 2002), examined the effects of alcohol consumption on PAD. Both studies used the ankle-to-brachial index (<0.90), which compares BP measured at the ankle with BP measured at the upper arm, as a measure of PAD.

The Strong Heart Study enrolled only American Indian subjects. After controlling for other factors, “current alcohol drinking” in this cohort was inversely associated with PAD prevalence in men and women (Fabsitz et al. 1999). Because more specific information was not available about levels or amounts of alcohol consumption associated with what the researchers called “current alcohol drinking” and only American Indians were included, it is difficult to generalize these findings.

The Rotterdam Study was designed to prospectively evaluate the occurrence of chronic diseases in an aging population (Vliegenthart et al. 2002). Their findings suggest that moderate alcohol consumption had no effect on PAD in nonsmoking men (Vliegenthart et al. 2002). In contrast, nonsmoking women had a significantly lower risk of PAD compared with nondrinking women for all levels of alcohol consumption, with the lowest risks found in women consuming 20 g/day of alcohol, or <2 drinks (odds ratio 0.32 [95% CI 0.11–0.91]).

Evaluating results from the Cardiovascular Health Study, Mukamal and colleagues (2008) found that drinking 1 to 13 drinks/week was associated with a lower risk of hospitalization related to lower-extremity arterial disease (defined as the diagnosis of atherosclerosis of native arteries of the extremities or peripheral vascular disease) in older adults (mean age >70) compared with former and nondrinkers. However, older adults who consumed >14 drinks/week did not experience the same reductions in PAD risk. In the latter study, analyses were adjusted for gender and many other confounding variables, but men and women were not analyzed separately.

Xie and colleagues (2010) conducted a large cross-sectional study of Chinese men ages ≥35 (*n* = 14,618). They found an inverse association between a key indicator of heart disease (i.e., ankle-to-brachial artery index >0.9) and alcohol consumption of <60 g/day, or about 4 drinks. Drinking ≥60 g/day was associated with a decrease in the ankle-to-brachial artery index, indicating greater risk for PAD. In that same study, no effect or relationship was found between any level of alcohol consumption and the ankle-to-brachial artery index in women.

Garcia-Diaz and colleagues (2011) examined the effects of alcohol consumption among PAD patients (*n* = 1,073). The PAD subjects chosen had either cramping pain in the leg brought on by exertion (typically caused by obstruction of the arteries) and known as intermittent claudication, with an ankle-to-brachial index <0.9; or they had previous vascular intervention or limb amputation for PAD. These subjects were enrolled in the Factores de Riesgo y ENfermedad Arterial (FRENA) registry, which was designed to examine the natural history of PAD in subjects (men and women) with a mean age >62. Over an average followup of 13 months, there were no differences between alcohol consumers and nonconsumers in PAD outcomes. However, mortality rates were greater in nonconsumers compared with alcohol consumers. The FRENA registry found mortality benefits across all different levels of alcohol consumption measured (e.g., <20 g/day, 21 to 61 g/day, and >60 g/day). However, as the authors note, only 19 percent of the subjects in the FRENA registry reported consuming >60 g/day of alcohol, limiting the generalizability of these findings.

As a result, existing data in this area suggest either a weak positive or small inverse relationship between low-to-moderate alcohol consumption (e.g., 1 to 13 drinks/week) and PAD prevalence in men. Compared with other studies, Xie and colleagues (2010) reported a greater “protective” threshold of alcohol consumption (<60 g/day) for PAD in men. However, in that study, the mean age of both male and female participants was ~50 years, nearly 15 to 20 years younger than in other studies (Mukamal et al. 2008; Vliegenthart et al. 2002). Findings are less clear for women, with some studies reporting a moderate inverse effect (Vliegenthart et al. 2002) and others detecting none at all (Xie et al. 2010).

In terms of specific PAD complications, Garcia-Diaz and colleagues (2011) found no differences in PAD outcomes between alcohol consumers and nonconsumers who had PAD. However, varying levels of daily alcohol consumption were associated with lower CV mortality and overall mortality rate (Garcia-Diaz et al. 2011). Larger prospective studies are required to define the association between dose, frequency, duration, and pattern of alcohol use and peripheral vascular disorders more precisely, so that researchers may formulate specific recommendations for men and women with PAD across populations.

## Mechanisms Related to Alcohol’s Positive and Adverse Effects on CV Conditions

Many of the CV conditions outlined above share the pathophysiologic process of atherosclerosis and inflammation. Therefore, alcohol may exert its protective or enhancing effects on these conditions by modifying three broad categories of mechanisms: risk factors (e.g., lipid profiles, carotid intima-medial thickness [cIMT], and insulin sensitivity), hemostatic factors (e.g., fibrinogen levels and platelet reactivity), and inflammation. In addition, and specific to CHD, alcohol consumption may modulate ischemia–reperfusion mechanisms as blood flow is restored to tissues after oxygen deprivation. Several of these potential mechanisms are briefly reviewed below.

## Risk Factors

One common risk factor for CV disease is the composition of the lipids found in the blood, and the effects of alcohol consumption on lipid profiles have been extensively studied. Many researchers have found that alcohol intake increases HDL cholesterol (HDL-c) levels, HDL (“good cholesterol”) particle concentration, apolipoprotein A-I, and HDL-c subfractions (Gardner et al. 2000; Muth et al. 2010; Vu et al. 2016). Findings have been equivocal for other lipids, such as low-density lipoprotein cholesterol (LDL-c) (the estimated amount of cholesterol within LDL particles, or “bad cholesterol”) and triglyceride levels (Rimm et al. 1999; Volcik et al. 2008; Waskiewicz and Sygnowska 2013). High triglyceride levels in the blood stream have been linked to atherosclerosis and, by extension, increased risk of CHD and stroke. However, in a recently conducted Mendelian randomization study, Vu and colleagues (2016) reported that low-to-moderate alcohol consumption reduced triglyceride and LDL-c and increased HDL-c, in particular the HDL2-c subfraction. Interestingly, the researchers found a nonlinear effect of alcohol consumption on HDL2-c levels. This supports the findings from other studies that the alcohol-induced changes in HDL-c do not fully account for the lower risk of CHD in moderate alcohol drinkers (Mukamal 2012).

Other risk factors that are surrogate markers of atherosclerosis and future CHD events, such as cIMT, also have been examined. The relationship between alcohol consumption and cIMT was inconsistent. Mukamal and colleagues (2003*b*) reported that older adults (age >70) consuming 1 to 6 drinks/week had lower cIMT compared with abstainers and those having ≥14 drinks/week. This is in contrast to results from other large population-based studies of older (age >70) (Zureik et al. 2004) or middle-aged (ages 45–64) (Demirovic et al. 1993; Djousse et al. 2002) adults, which did not find a relationship between level of alcohol intake and cIMT.

Some reports suggest that low-to-moderate alcohol consumption is associated with favorable effects in insulin sensitivity and glucose metabolism, key risk factors in the development of diabetes (Greenfield et al. 2005).^3^ Randomized placebo-controlled trials conducted with nondiabetic postmenopausal women showed that 2 drinks per day significantly lowered insulin levels during fasting and after meals and increased insulin sensitivity (Davies et al. 2002). Increased insulin sensitivity, which is the opposite of insulin resistance, is associated with a reduced risk for the development of type 2 diabetes and CHD.

## Hemostatic Factors

Alcohol consumption can be associated with both a favorable hemostatis/coagulation profile as well as an adverse one (Salem and Laposata 2005). Several epidemiologic and randomized controlled studies have found alcohol consumption decreases coagulation factors such as fibrinogen, which is a CV risk marker at elevated levels (Mori et al. 2015; Rimm et al. 1999). In addition to being essential to the coagulation cascade, fibrinogen also may play a proinflammatory role in the development of certain CV diseases, including vascular wall disease and atherosclerosis (Davalos and Akassoglou 2012). Findings from a meta-analysis of 42 studies by Rimm and colleagues (1999) suggested that 30 g of alcohol/day (2 standard drinks) was associated with a 7.5 mg/dl (−17.7 to 32.7) decrease in fibrinogen concentration. Similarly, the results from the small randomized crossover trial by Mori and colleagues (2015) found that women consuming alcohol (146 to 218 g/week, ~2 to 3 standard drinks/day) for 4 weeks showed a 14 percent reduction in fibrinogen levels.

Platelets and their role in clotting also affect CV disease. Altered platelet responses (e.g., increased platelet activation/aggregation) leads to blood-clot formation (or thrombosis) in certain CV conditions. Anticlotting therapies are therefore the cornerstone of managing acute coronary syndromes. Not surprisingly, alcohol consumption has complex and varying effects on platelet function. Studies using different methodologies have shown that low-to-moderate alcohol consumption decreases platelet activation and aggregation in certain cases—for example, in response to certain physiologic stimuli such as adenosine 5′-diphosphate (Salem and Laposata 2005). On the other hand, significant daily alcohol consumption increases platelet aggregation and reactivity. Infection or other stressful events also can lead to immune-triggered platelet production, a condition called rebound thrombocytosis, which may occur immediately after withdrawal from both heavy and one-time heavy (binge) drinking (Numminen et al. 1996). Although highly individualized and dose dependent, alcohol use also can increase bleeding time (i.e., taking longer to develop a clot)(Salem and Laposata 2005).

## Inflammation

The effects of alcohol consumption on inflammation are twofold. Lower doses are associated with reduced inflammation, as indicated by markers such as C-reactive protein and certain interleukins. Conversely, higher levels induce oxidative stress and a wide variety of inflammatory markers. As reviewed below, oxidative stress in particular is likely a key event in the development of alcoholic cardiomyopathy (discussed in “Acute and Long-term Effects of Alcohol on the Myocardium”). Data from numerous types of research studies show that alcohol may alter levels of antioxidant enzymes and stimulate oxidative damage, and it may therefore be involved in the pathogenesis of many types of alcohol-induced diseases (Ceni et al. 2014; Piano and Phillips 2014).

## Ischemic Preconditioning

Another mechanism underlying the cardioprotective effects of low-to-moderate alcohol consumption and CHD in particular may be related to a phenomenon known as ischemic preconditioning, which produces resistance to the loss of blood supply (and oxygen) to organs or tissues. If the blood supply is impaired briefly (usually for <5 minutes) and then restored so that blood flow resumes, and the process is repeated two or more times, the cells downstream of the tissue or organ are protected from a final ischemic insult, when the blood supply is cut off. In the heart, this would protect the heart muscle (myocardium) from subsequent, more prolonged episodes of restricted blood flow (ischemia) followed by injury when that blood flow returns to the heart (called reperfusion injury or ischemia–reperfusion injury; Veighey and Macallister 2012). Ischemic preconditioning results in smaller infarct sizes, fewer and less severe arrhythmias, and prevention of endothelial cell dysfunction (Veighey and Macallister 2012). During the ischemic phase, the flow of oxygen and nutrients to the tissues is reduced, most significantly to the heart, brain, and kidneys. In contrast, during the reperfusion phase, despite restoration of blood flow, a series of dysfunctional biochemical and metabolic changes are initiated that lead to extensive accumulation of ROS. ROS induce a number of changes. One is the opening of the mitochondrial permeability transition pore, which is formed in the mitochondria during ischemic incidents, contributing to reperfusion injury and cell death. Others include recruitment of neutrophils (white blood cells that are among the first inflammatory cells to respond during inflammation) and dysfunction of the sarcoplasmic reticulum, which can affect calcium ion storage and release into muscle fibers.

Alcohol may affect various mechanisms implicated in ischemic preconditioning. Among these is the activation of mitogen-activated protein kinases (MAPK) signaling cascades. MAPKs are activated in response to stressful stimuli and help regulate apoptosis. There also is desensitization of the mitochondrial permeability transition pore, which can mitigate ischemia–reperfusion injury (Walker et al. 2013). In addition, alcohol may attenuate ischemia–reperfusion injury by activating protein kinase C epsilon (PKCɛ) (Walker et al. 2013). Activation of PKCɛ may protect the myocardium against ischemia–reperfusion injury by stimulating the opening of mitochondrial ATP-sensitive potassium channels. This in turn prevents the opening of the mitochondrial permeability transition pore (Walker et al. 2013).

**Figure 3:**
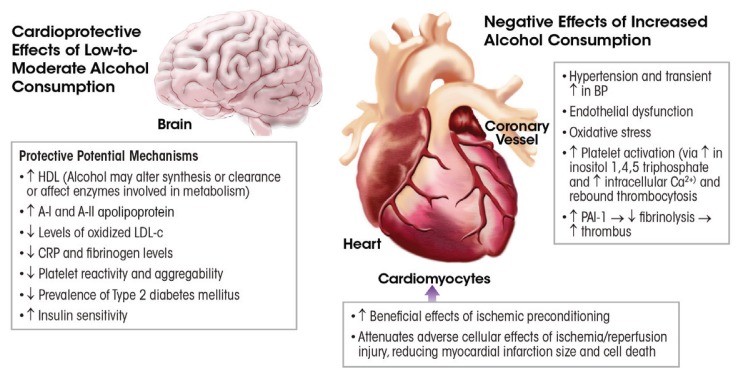
Mechanisms related to the positive and adverse effects of alcohol on cardiovascular conditions, such as coronary heart disease and stroke as well as cardiomyopathy. Different mechanisms may be in effect depending on the dose, duration, and pattern of alcohol consumption. NOTE: BP = blood pressure, Ca^2+^ = calcium, CRP = C-reactive protein, DM = diabetes mellitus, HDL = high-density lipoprotein, LDL = low-density lipoprotein, PAI-1 = plasminogen activator inhibitor-1. SOURCE: Adapted from Krenz and Korthuis 2012.

Figure 3 summarizes the potential mechanisms underlying the cardioprotective and adverse effects of alcohol consumption. One or more mechanisms may be in effect and/or may cancel out another. This area of research was briefly outlined here; more comprehensive reviews on these mechanisms are available (Krenz and Korthuis 2012; Mathews et al. 2015).

## Impact of Drinking Patterns and Types of Alcoholic Beverages on Risk

Drinking patterns, and in particular a binge pattern of drinking and higher frequency of binge drinking, are associated with a heightened risk of CV conditions such as HTN, stroke, and MI, as well as sudden death or increased mortality after MI (Leong et al. 2014; Marques-Vidal et al. 2001; Mukamal et al. 2005; Sundell et al. 2008; Wannamethee and Shaper 1992). In a systematic review (*n* = 37 studies), Feigin and colleagues (2005) found that excessive ethanol intake (>150 g ethanol/week) was associated with a doubled risk of subarachnoid hemorrhage. The latter findings may relate to the overall large quantity of alcohol consumed (~12 standard drinks/week) rather than a binge pattern.

Binge drinking in younger individuals also may increase the risk of stroke. Haapaniemi and colleagues (1996) found that acute intake on weekends and holidays of >40 g ethanol was significantly associated with cerebral infarction within 24 hours in young (ages 16–40) and middle-aged (ages 41–60) subjects. One possible mechanism for the binge-associated increased stroke risk is HTN. However, at least in younger people, HTN prevalence is low, suggesting that other mechanisms may be involved.

It has been debated whether beverage type has differential effects. Some investigators have suggested that drinking wine may offer more protection against CV disease because it contains polyphenols, such as resveratrol and flavonoids, which are micronutrients with antioxidant activity (Tangney and Rasmussen 2013). However, among studies designed to examine the influence of beverage type, no differences have been found in CV disease outcomes or biologic markers, such as HDL-c (Mukamal et al. 2003*a*; Volcik et al. 2008). Differential associations of CV risk with certain beverage types such as wine instead have been attributable to other lifestyle factors (e.g., increased physical activity) or drinking with meals (Malarcher et al. 2001). The findings from INTERHEART, in which “any alcohol use” had no cardioprotective effects in certain populations, such as in people of South Asian ethnicity who live in South Asia (e.g., India, Bangladesh, Nepal), led to speculation about beverage type, beverage quality, and drinking pattern as important mediators (Leong et al. 2014).

Finally, in studies of people from certain Eastern European countries, investigators have failed to find a cardioprotective effect with any level of ethanol consumption (Britton and McKee 2000). This suggests that alcoholic beverage type may be an important mediator, because in countries such as Russia, spirits are the alcoholic beverage of choice. However, the negative associations between alcohol consumption and CV outcomes in these countries also may relate to pervasive patterns of binge drinking (Leon et al. 2009).

## Acute and Long-term Effects of Alcohol on the Myocardium

### Acute Effects

The acute effects of alcohol on the myocardium include a weakening of the heart’s ability to contract (negative inotropic effect). Data from isolated papillary and heart muscle cell (myocyte) experiments demonstrate that acute physiologic intoxicating doses of alcohol (80 mg% to 250 mg%) can have a negative inotropic effect (Danziger et al. 1991; Guarnieri and Lakatta 1990). These effects also may involve an irregular and often very fast heart rate (arrhythmia) during which the heart’s upper chambers (atria) contract chaotically out of coordination with its lower chambers (ventricles), known as atrial fibrillation, or (rarely) sudden cardiac death.

Investigators have used a variety of noninvasive tests to evaluate the acute effects of alcohol consumption on myocardial function and hemodynamics in healthy humans. As with isolated animal heart experiments, some investigators have found that acute alcohol exposure (blood alcohol levels 40 to 110 mg%) depresses myocardial systolic function in humans (Delgado et al. 1975; Lang et al. 1985; Timmis et al. 1975). However, these changes were transient, with small changes from baseline. For example, in one study, the ejection fraction decreased by 4 percent after alcohol consumption (Delgado et al. 1975). In another study by Lang and colleagues (1985), however, the researchers noted a decrease in the maximum pressure developed by a ventricle at any given left ventricular volume, plotted as the end-systolic pressure dimension slope, as well as a decrease in the rate-corrected velocity of left-ventricular fiber shortening—and cardiac output was increased. Most likely, the decrease in contractility was offset by corresponding decreases in afterload (end-systolic wall stress), systemic vascular resistance, and aortic peak pressure, which maintained cardiac output.

Other researchers have reported that acute alcohol consumption resulting in blood alcohol levels of 100 to 120 mg% exerted no effect on cardiac performance (Blomqvist et al. 1970; Child et al. 1979; Kupari et al. 1983*a*,*b*). It is important to note that most studies were performed >30 years ago with young subjects (mean age 23–35) and with small sample sizes (*n* = 4–12). As a result, whether or how these findings generalize to older healthy people and those with CV disease is unknown. However, in an elderly community- based population (i.e., the Atherosclerosis Risk in Communities Study, mean age at time of study 74–76 years), Gonçalves and colleagues (2015) examined the effects of different levels of weekly alcohol consumption on alterations in cardiac structure and function. These investigators found increasing amounts of alcohol were associated with mild alterations in cardiac structure and function, which were greater in women. In the United States, it is estimated that by 2060 there will be 98 million adults age >65, more than twice the number in 2014 (Administration on Aging 2014). In addition, recent research indicates that this generation will potentially consume alcohol at higher rates than previous generations (Barry and Blow 2016). Consequently, more research may be necessary to better understand the effects of alcohol consumption on the CV systems of older adults.

Certain arrhythmias, such as atrial fibrillation, may be the most serious consequence of consuming large amounts of alcohol, and in particular binge drinking. Larsson and colleagues (2014) have reported that binge drinking (defined by these researchers as having more than 5 drinks on a single occasion) was associated with an increased risk of new-onset atrial fibrillation. Atrial fibrillation is one of the most common arrhythmias and is strongly associated with adverse CV events, such as stroke (Conen et al. 2011). Results from retrospective studies enrolling adults ages 40–60 also have linked binge drinking to a heightened risk of sudden death (Wannamethee and Shaper 1992).

### Long-term Effects

Alcoholic cardiomyopathy (ACM) is a heart-muscle disease found in individuals with a history of long-term heavy alcohol consumption. It is characterized by a dilated left ventricle (LV), normal or reduced LV wall thickness, increased LV mass, and (in advanced stages) a reduced LV ejection fraction (<40 percent) (Piano and Phillips 2014). There are no specific immunohistochemical or immunological biomarkers or other criteria for an ACM diagnosis (Piano and Phillips 2014). Therefore, a key factor in diagnosing ACM is a long-term history of heavy alcohol abuse without CHD or other cardiac conditions such as inflammation of and damage to the myocardium, known as myocarditis.

ACM’s exact prevalence remains elusive. The proportion of cardiomyopathy cases attributable to alcohol abuse has ranged from 23 to 40 percent (Piano and Phillips 2014). Recently, Guzzo-Merello and colleagues (2015) reported that, among 282 patients with a dilated cardiomyopathy phenotype, 33 percent had ACM. Both men and women can develop ACM. However, some reports indicate that alcohol-dependent women develop ACM after consuming less alcohol over a shorter period than do age-matched alcohol-dependent men (Fernández-Solà et al. 1997; Urbano-Marquez et al. 1989).

In humans, the exact amount and duration of alcohol consumption associated with development of ACM remains unknown. The point at which alcohol-induced abnormalities appear over the course of a person’s lifetime drinking also is not well established and is highly individualized. This suggests either protective or adverse interaction effects of genetic or lifestyle factors (Piano and Phillips 2014). Among ACM patients (*n* = 94) referred to a heart failure and heart transplant unit, Guzzo-Merello and colleagues (2015) found the mean alcohol consumption was ~11 drinks/day for at least 20 years, with most patients consuming slightly less (6 to 8 drinks/day). The researchers reported a mean age for these ACM patients of ~50, and more than half were current cigarette smokers.

ACM patients can present with either diastolic or systolic dysfunction and may or may not have symptoms of heart failure. When these patients are treated with standard heart failure therapies, they have good clinical outcomes and reduced mortality rates. Factors associated with poor outcomes (e.g., greater mortality or transplantation) included a history of atrial fibrillation; an electrocardiogram QRS width >120 milliseconds (ms), compared with the normal range of 70 to 100 ms; and not being treated with beta-blockers or digoxin (Guzzo-Merello et al. 2015).

**Figure 4:**
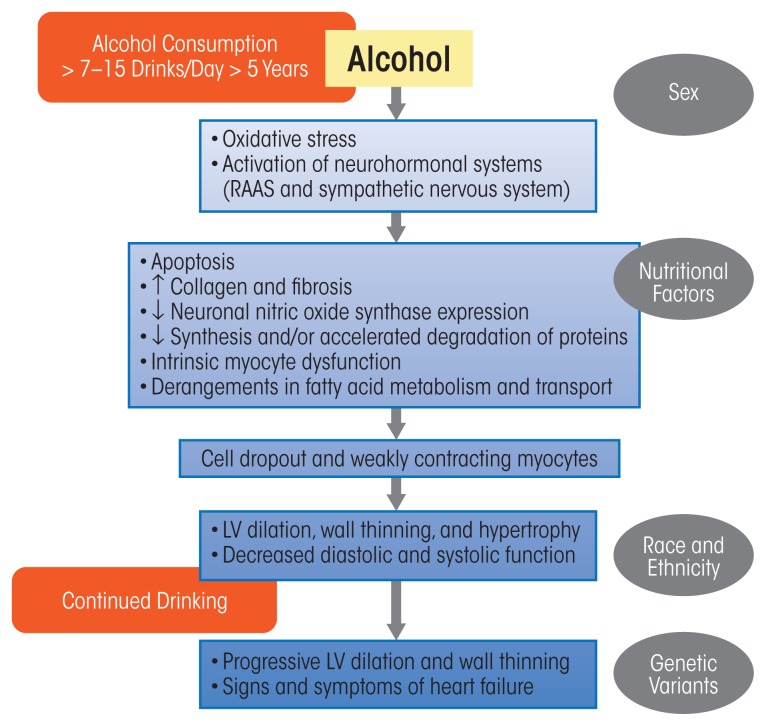
Pathophysiologic schema for the development of alcoholic cardiomyopathy (ACM). As noted in the text, the exact amount and duration of alcohol consumption that results in ACM in human beings varies. The exact sequence of the development of ACM remains incompletely understood. Data from animal models and human beings with a history of long-term drinking suggest that oxidative stress may be an early and initiating mechanism. Many cellular events, such as intrinsic myocyte dysfunction, characterized by changes in calcium homeostasis and regulation and decreased myofilament sensitivity, can come about due to oxidative stress. Variables in gray ovals represent potential mediating factors. NOTE: LV = left ventricle, RAAS = renin–angiotensin–aldosterone system. SOURCE: Adapted from Piano and Phillips 2014.

Long-term heavy alcohol consumption induces adverse histological, cellular, and structural changes within the myocardium. As with other alcohol-induced pathologies, mechanisms contributing to ACM include oxidative stress, apoptotic (programmed) cell death, impaired mitochondrial bioenergetics and stress, derangements in fatty acid metabolism and transport, and accelerated protein breakdown; these will be discussed in the following sections. These mechanisms contribute to the myocyte cellular changes that lead to intrinsic cell dysfunction, such as sarcoplasmic reticular dysfunction and changes in intracellular calcium handling and myocyte loss. However, modulatory influences related to drinking patterns, genetic susceptibility, nutritional factors, ethnicity, and gender also many play a role (Piano and Phillips 2014) (figure 4).

## Oxidative Stress and Apoptosis: Linked Mechanisms

### Oxidative Stress

In examining alcohol-induced pathologies, other researchers have suggested three potential ways in which alcohol exposure can lead to excess free-radical generation and oxidative stress: ethanol metabolism, ethanol effects on antioxidant proteins and antioxidant enzymes, and activation/alteration in neurohormonal systems (table 2) (Fernández-Checa et al. 1998; Piano and Phillips 2014). At least in the myocardium, many adverse cardiac intracellular effects found after chronic alcohol consumption can be attributed to oxidative stress:

- Myocyte loss and disarray;
- Sarcoplasmic reticulum dysfunction, which can lead to systolic dysfunction as well as a thickening of the heart muscle that can make ventricles larger, known as cardiac hypertrophy (Bing et al. 1974; Segel et al. 1981);
- Changes in handling of intracellular calcium ions (Zhang et al. 2014);
- Depressed or disturbed mitochondrial function (Pachinger et al. 1973; Segel et al. 1981; Weishaar et al.1977);
- Decreased myofibrillar ATPase activity, which affects muscle contraction (Hastillo et al. 1980; Segel et al. 1975);
- Decreased myofibrillar calcium sensitivity, which affects contractile force generation in the heart (Piano et al. 1999);
- Contractile protein fragmentation and disarray (Jiang et al. 2012; Tsiplenkova et al. 1986; Urbano-Marquez et al. 1989); and
- Fatty acid accumulation within intracellular organelles, which can include atypical storage of fat in heart tissue that can lead to dysfunction (Beckemeier and Bora 1998; Hu et al. 2013).

In various biologic systems, oxidative stress can be measured or inferred by several biologic indexes. These can include measurement of antioxidant enzymes (e.g., catalase or glutathione peroxidase) or scavenging proteins (e.g., glutathione), oxidative damage (e.g., increase or presence of protein carbonyl and conjugated diene levels), or measurement of circulating oxidative products (e.g., isoprostanes) (Dalle-Donne et al. 2006; Montuschi et al. 2004). Collectively, data from human and animal models suggest that alcohol may alter important components of the antioxidant defense system, such as levels of antioxidant substrates (e.g., decreased glutathione levels) or levels or activity of antioxidant enzymes (e.g., decreases in catalase or superoxide dismutase) (Edes et al. 1986, 1987; Ribiere et al. 1992; Vendemiale et al. 2001) and/or lead to increased levels or accumulation of other markers of oxidative stress (such as conjugated dienes, protein carbonyls, or 3-nitrotyrosine) (Edes et al. 1986, 1987; Ribiere et al. 1992; Tan et al. 2012; Vendemiale et al. 2001; Zhang et al. 2014).

Evidence of oxidative stress is found after short periods of alcohol consumption (2 to 18 weeks), at least in animal models. These data suggest that antioxidant defense mechanisms that attempt to protect the heart against oxidative damage appear to be initiated soon after drinking alcohol. Also, as noted below, data from other studies demonstrate the protective role of administered antioxidants, such as a synthetic compound that mimics the native superoxide dismutase enzyme, called a superoxide dismutase mimetic. This suggests a direct or indirect role for ethanol-mediated oxidative stress in the heart (Jiang et al. 2012; Tan et al. 2012).

Data from transgenic animal models and pharmacologic approaches strongly support a role for ethanol-induced oxidative stress in CV disease. Using both pharmacologic and transgenic approaches, Tan and colleagues (2012) showed that administering an ROS scavenger (a superoxide dismutase mimetic) to mice receiving a diet high in ethanol for 2 months significantly reduced nitrative damage (i.e., decreased 3-nitrotyrosine accumulation, a marker of decreased cell damage and inflammation). In addition, there was no evidence of nitrative damage in mice bred to disrupt (i.e., knock out) the gene for angiotensin I receptor (AT1-KO) that had been given ethanol for a similar length of time (Tan et al. 2012). Both experimental approaches also prevented accumulation of ethanol-induced scarring (collagen and fibronectin); apoptotic cell death; and changes in the size, shape, and function of the heart after injury to heart muscle (ventricular remodeling).

Other researchers have used genetic approaches (i.e., transgenic animals) to prevent ethanol-induced oxidative stress. One approach included overexpression of proteins such as insulin-like growth factor (IGF-1), which stimulates growth and cell proliferation and has antiapoptotic effects (see Zhang et al. 2014). In contrast to control mice, the IGF-1–expressing animals exhibited no evidence of changes in expression of antioxidant enzymes (i.e., superoxide dismutase-1) or any decreases in contractile function after 16 weeks of ethanol consumption. The findings suggest a protective effect of overexpression of IGF-1 in the transgenic animals (Zhang et al. 2014).

## Apoptosis

Apoptosis also may be an important mechanism in ACM and a consequence of oxidative stress. Studies have found evidence of apoptosis in humans with ACM and in animal ACM models. Fernández-Solà and colleagues (2006) evaluated apoptosis in the hearts of adults with long-term alcoholism (*n* = 19, drinking for 26 years), adults with long-standing hypertension (*n* = 20), and those with no known disease (control subjects, *n* = 7). Apoptosis as evidenced by increased protein expression of two key proteins—one that promotes apoptotic cell death (i.e., BAX) and one that inhibits it (i.e., BCL-2)—was significantly higher in both the alcoholic subjects and in the hypertensive subjects, compared with control subjects (Fernández-Solà et al. 2006). Moreover, apoptosis was of a similar magnitude in the alcoholic and the hypertensive subjects. More recent findings from this research group corroborate that apoptosis occurs in humans who have a long history of heavy alcohol consumption (Fernández-Solà et al. 2011).

## Mitochondrial Dysfunction and Changes in Mitochondrial Bioenergetics

Researchers have found evidence of mitochondrial dysfunction or impaired bioenergetics related to alcohol consumption. This is not surprising, because mitochondria are a major target for free-radical injury. Dysfunctional mitochondria are less efficient, can become a source of ROS, and are more likely to initiate apoptosis (Marzetti et al. 2013).

Histological studies published several decades ago reported evidence of mitochondrial injury, such as mitochondrial enlargement and disorganization, increased number of mitochondria, mitochondriosis (small mitochondria closely packed together), and an increase in lysosome-like structures that break down biomolecules in myocardial postmortem biopsy samples from people with a long-term history of heavy alcohol consumption (Hibbs et al. 1965; Tsiplenkova et al. 1986; Vikhert et al. 1986).

More contemporary studies have not found evidence of mitochondrial injury in biopsy samples from long-term alcohol drinkers (Miró et al. 2000). Differences among results from human studies may relate to small sample sizes, duration of drinking, and degree of myocardial dysfunction. In the Miró study, alcohol drinkers also had been receiving pharmacologic treatments such as beta-adrenergic blocking agents that reduce blood pressure and also may have antioxidant effects.

Changes in mitochondrial function have been reported from a number of animal studies in different species, under various alcohol consumption paradigms (ethanol in water or liquid diet), and after variable durations of chronic ethanol consumption (6 weeks to 6 months). Through the process of oxidative phosphorylation, the mitochondria generate ~90 percent of cellular ATP. Common findings in alcohol studies from the 1970s and early 1980s included decreases in mitochondrial indices that reflected mitochondrial state III respiration, or ADP-stimulated respiration (Pachinger et al. 1973; Segel et al. 1981; Williams and Li 1977). The latter changes in these indices could be brought about by ethanol-induced imbalances in the reducing equivalents nicotinamide adenine dinucleotide (NAD) and nicotinamide adenine dinucleotide hydrogen (NADH), an important chemical pathway involved in oxidative stress. In cardiomyocyte mitochondria as well as other mitochondrial types, such imbalances could lead to further decreases in cellular respiration and oxidative phosphorylation.

More recent studies have confirmed that 4 to 16 weeks of ethanol consumption was associated with mitochondrial dysfunction. This was evidenced by decreased myocardial ATP content levels, changes in the mitochondrial membrane potential, and decreases in cytochrome oxidase activity in conjunction with decreased myocardial contractility (e.g., decreased ejection fraction and fractional shortening) (Hu et al. 2013; Laurent et al. 2014). Although the connection is still speculative, this reduction in ATP synthesis may be enough to depress important intracellular functions that support heart health, such as sarcoplasmic reticulum uptake of calcium ions and cross-bridge cycling in muscle contraction. Prolonged ethanol consumption also may decrease expression of several types of mitochondrial proteins, such as NADH dehydrogenase, isocitrate dehydrogenase, and long-chain-specific acyl-CoA dehydrogenase, as well as proteins within the citric acid cycle (Fogle et al. 2010). This kind of mitochondrial dysfunction, including decreased expression of some of these proteins, is integral to cardiac ischemic–reperfusion injury, which occur routinely with MI—the most common incident of CV disease, itself the number-one cause of death (Tompkins et al. 2006).

## Derangements in Fatty Acid Metabolism and Transport

Derangements in fatty acid metabolism and transport and formation of fatty acid ethyl esters (FAEEs) also have been implicated in ethanol-induced cell injury. FAEEs can be formed in the body during ethanol metabolism, when ethanol reacts with fatty acids or triglycerides. FAEEs can attach to mitochondria and disrupt mitochondrial function. Lange and Sobel (1983) were the first to identify an increase in FAEE content in postmortem myocardium samples obtained from those subjects who routinely had used large amounts of alcohol (*n* = 2) and who had a history of recent alcohol intoxication (*n* = 4).

The idea that FAEEs are involved in ACM pathogenesis and are cytotoxic is supported by the fact that increased tissue levels of FAEE are considered the mechanism underlying cell death induced using a procedure to control and prevent recurrence of cardiac arrhythmias (i.e., septal ablation) (Yoerger et al. 2006).

Other researchers have confirmed in animal models that long-term ethanol consumption can also affect long-chain fatty acid (LCFA) uptake, as well as increased expression of the genes encoding for proteins involved in the formation of triglycerides from free fatty acids and glycerol, or triglyceride esterification, and in LCFA transporters (Hu et al. 2013).^4^

## Accelerated Protein Degradation

Long-term alcohol use decreases myocardial protein expression and synthesis and accelerates protein degradation in the myocardium (Lang et al. 2005). This in turn disrupts myocardium function, including contraction and relaxation of the cardiac walls, impairing the heart’s ability to pump blood. Using mass spectrometric–based proteomic analysis in an animal model, Fogle and colleagues (2010) found that long-term alcohol consumption was associated with decreases of 30 to 54 percent in cell-scaffolding proteins (myofibrillar α-myosin and actin) and mitochondrial proteins (mitochondrial dehydrogenases and electron transport proteins), glycolytic enzymes (glycogen phosphorylase and alpha-enolase), and fatty acid metabolism proteins (fatty acid transport protein and LCFA acyl-CoA ligase). These investigators also found decreases in peroxiredoxin 5, antioxidant protein 2, and glutathione transferase 5—important antioxidant enzymes whose cardiovascular protective functions still are not fully understood. For example, some findings suggest an inverse role between peroxiredoxin 5 and stroke severity. During a severe stroke, peroxiredoxin 5 is consumed and its production impaired (Kunze et al. 2014).

Other ethanol-induced changes may be related to enzymes that modulate protein synthesis and/or breakdown (e.g., ubiquitine-ligases). Several reports suggest that ethanol-induced decreases in myocardial protein synthesis may be mediated in part by decreased activity of an enzyme called mammalian (or mechanistic) target of rapamycin (mTOR) (Lang and Korzick 2014; Vary and Deiter 2005; Vary et al. 2008). mTOR regulates cell growth, proliferation, motility, and survival; protein synthesis; and transcription (Donohue 2009). Decreases in mTOR activation may play a role in reduced myocardial protein synthesis, ventricular wall thinning, and dilation.

Alterations in protein physiology and content also can result from accelerated protein degradation. The normal destruction of molecules and cell organelles (called autophagy) may be especially important in triggering ACM. Autophagy is performed by lysosomes and involves a breakdown (catabolism) of unnecessary or damaged proteins in the cell. This mechanism also is essential to cell and organism survival during stress and nutrient deprivation. Under the latter conditions, autophagy helps generate and recycle carbons and amino acids through degradation of large (macromolecular) cellular constituents. However, as with other CV pathological conditions, such as heart failure and cardiac hypotrophy, there is evidence of increased autophagy with chronic alcohol consumption. Guo and colleagues (2012) studied autophagy in mice with and without an overexpression of the enzyme alcohol dehydrogenase to show that 8 weeks of ethanol consumption was associated with increased myocardial markers of autophagy, such as autophagy-related 7 protein.

Increased autophagy as a possible mechanism underlying the adverse myocardial effects of ethanol is intriguing. This is especially true in light of the relationship between a sensor of stress (mTOR) and nutrient deprivation and how essential autophagy is to cell survival. Activation of mTOR leads to inhibition of autophagy. As noted above, chronic alcohol exposure leads to a decrease in mTOR activity, which corresponds to increased markers of autophagy (Lang and Korzick 2014). The autophagy pathway also is rapidly upregulated during ATP depletion, mitochondrial dysfunction, and oxidative stress. Ethanol-mediated increases in autophagy therefore may be an important mechanism underlying the adverse myocardial effects of ethanol.

**Figure 5:**
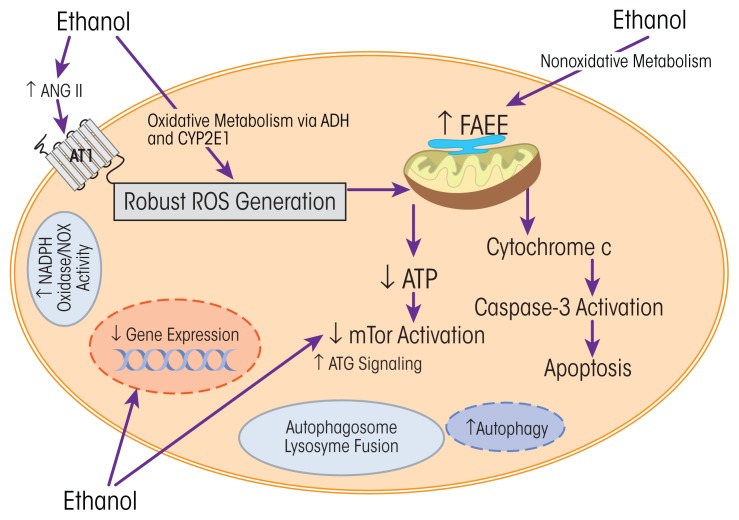
Summary of potential cellular changes related to ethanol. Ethanol-induced changes may be related to oxidative or nonoxidative pathways of ethanol metabolism. More than one mechanism may be activated and may lead to the multitude of ethanol-induced changes in cellular proteins and cell function. As reviewed in the text, data from pharmacologic and transgenic approaches revealed an important role for oxidative stress and the hormone angiotensin II. NOTE: Ang II = angiotensin II, ATG = atrogin, ATI = angiotensin I receptor, ATP = adenosine triphosphate, CYP2E1 = cytochrome P450 2E1, FAEE = fatty ethyl esters, mTOR = mammalian (or mechanistic) target of rapamycin, NADPH oxidase/NOX = nicotinamide adenine dinucleotide phosphate-oxidase, ROS = reactive oxygen species. SOURCE: Adapted from Piano and Phillips 2014.

Some of the potential cellular changes related to ethanol consumption reviewed above are illustrated in figure 5. More than one cellular event may be happening at the same time, and, as with other chronic health conditions, the relevant mechanisms may be synergistic and interrelated.

## Summary and Future Directions for Research

Alcohol consumption remains a major risk factor for global burden of disease (Rehm et al. 2009). Nearly all the data on humans exploring the relationship between alcohol consumption and CV risk—including some indications of potential CV benefits associated with low-to-moderate alcohol consumption— are derived from epidemiologic studies. Therefore, because there are no randomized controlled trials, health care professionals should not recommend alcohol consumption as a primary or secondary lifestyle intervention. Instead, clinicians should continue to recommend strategies such as a healthy diet and exercise. Adults who choose to drink can be encouraged to follow the alcohol consumption recommendations from NIAAA (table 3).

Data derived from systematic reviews and meta-analyses suggest that alcohol-dose and CV-health relationships differ for various CV conditions. For example, certain levels of alcohol consumption that lower risk for CHD may increase it for other CV conditions, such as stroke. In addition, data from studies using new research methods, including Mendelian randomization, suggest that the relationship between low-to-moderate alcohol consumption and cardioprotection merits more critical appraisal (Holmes et al. 2014).

Alcohol use remains, as Saitz (2015) has memorably noted, “no ordinary health risk.” Heavy daily alcohol consumption and binge drinking increase the risk of developing CV disease. The growing rate of binge drinking in the United States is a serious concern (Kanny et al. 2013). ACM, though not a leading cause of heart failure nationwide, can be associated with marked changes in cardiac function, symptoms, and poor quality of life. Susceptibility factors, such as gender, race/ethnicity, genetics, and socioeconomic factors, may influence alcohol’s positive and adverse health effects. When clinicians are counseling patients about alcohol consumption, they should consider all these factors, as well as any history of alcohol dependence. Of course, any advice about alcohol consumption and related health issues needs to be targeted for each patient.

This review suggests several recommendations for future research:

- Using direct biomarkers of alcohol, such as PEth, to corroborate self-report of alcohol consumption and distinguish between and among low, moderate, and heavy alcohol consumption.
- Examining the potential mediation of genetic, socioeconomic, and racial and ethnic factors within the alcohol–CV disease relationship, specifically regarding development of ACM.
- As suggested by Klatsky (2015), reviewing alcohol–medication interactions, considering the large number of antiplatelet agents, lipid-lowering, and antihypertensive therapies prescribed to people with CV conditions.
- Examining the CV effects related to alcohol use in young adults (ages 18–30), a group that consumes the most alcohol and binge drinks the most.
- Considering the growing number of older adults, more research is needed to better understand the effects of alcohol consumption on the CV systems of older populations.

## Figures and Tables

**Table 1:** INTERHEART Data, Patterns of Alcohol Use, and Odds Ratio (OR) for Myocardial Infarction (MI).

Pattern of Alcohol Use	OR (95%) for MI	*p* value
Any alcohol use within 12 months		
Unadjusted	0.92 (0.87–0.96)	<0.001
Adjusted (model 1)	0.81 (0.76–0.87)	<0.001
Adjusted (model 2)	0.87 (0.80–0.94)	0.001
Pooled*	0.84 (0.71–0.99)	0.04
Any alcohol use and risk of MI in subsequent 24 hours	1.0 (0.9–1.2)	0.70
≥ 6 Drinks and risk of MI in subsequent 24 hours	1.4 (1.1–1.9)	0.01

NOTE: Model 1 was adjusted for age (categorized as <45, 45–65, and >65 years), gender, geographic region, Dietary Risk Score, exercise, smoking, marital status, employment, education level, depression, stress at work or at home, financial stress, body mass index (BMI), and waist-to-hip ratio. Model 2 was adjusted as for Model 1 and for serum ratio of apolipoprotein B to apolipoprotein; total cholesterol, high-density lipoprotein, low-density lipoprotein, and triglyceride concentrations; and history of hypertension or diabetes mellitus. CI = confidence interval; MI = myocardial infarction; OR = odds ratio.

* P ooled effect estimates from conditional logistic regression were stratified by geographic region and adjusted for Dietary Risk score, exercise, smoking, marital status, employment, education level, depression, stress at work or at home, financial stress, BMI, and waist-to-hip ratio.

SOURCE: Used with permission from Leong et al. 2014.

**Table 2:** Potential Ethanol-Induced Sources of Reactive Oxygen Species.

**Ethanol Metabolism** An ↑ in the flux of reducing equivalents into the electron transport chain due to an ↑ in nicotinamide adenine dinucleotide production related to ethanol metabolism (↑ NADH/NAD+ ratio). An ↑ in cytochrome P450 2E1 metabolism of ethanol. An ↑ in alcohol dehydrogenase metabolism of ethanol and accumulation of acetaldehyde (leading to ROS formation and acetaldehyde adduct formation). Nonoxidative metabolism by fatty acid ethyl ester synthase and/or phospholipase D. **Ethanol Effects on Antioxidant Proteins and Antioxidant Enzymes** Alcohol-induced inhibition of transport proteins responsible for transporting glutathione from cytosol into the mitochondria (e.g., glutathione transport from cytosol into the mitochondria) and ↓ antioxidant enzyme levels and activity (e.g., superoxide dismutase). **Activation/Alteration in Neurohormonal Systems** Increased autoxidation of catecholamines. An ↑ in angiotensin II and norepinephrine levels.

NOTE: NAD = nicotinamide adenine dinucleotide, NADPH = nictotinamide adenine dinucleotide diphosphate, ROS = reactive oxygen species.

SOURCE: Used with permission from Piano and Phillips 2014.

**Table 3:** Drinking Levels Defined

The National Institute on Alcohol Abuse and Alcoholism defines low risk drinking for developing alcohol use disorder as: No more than 4 drinks on any single day and no more than 14 drinks per week for men age 65 or younger. No more than 3 drinks on any single day and no more than 7 drinks per week for women and men over the age of 65.

## Footnotes

1 The results of the NIAAA Wearable Alcohol Biosensor Challenge were announced May 19, 2016, at https://www.niaaa.nih.gov/research/challenge-prize.

2 *British Doctors Study.* Available at: http:/www.epi.umn.edu/cvdepi/study-synopsis/british-doctors-study/. Accessed December 13, 2016. *Physicians’ Health Study.* Available at: http://phs.bwh.harvard.edu/. Accessed December 13, 2016.

3 Greenfield and colleagues (2005) studied the effects of alcohol at meal time in a group of nonsmoking, healthy postmenopausal women. Each woman was given either no alcohol or 15 g of alcohol (1 standard drink) with either a low-carbohydrate or a high-carbohydrate, high-fat meal. The women’s metabolic measurements were then taken over the next 6 hours. The researchers found that the alcohol-drinking subjects (particularly those who were insulin sensitive) had higher insulin levels and a slower rise in glucose levels after a low-carb meal. They recommended confirming these results in younger women and in men, particularly since their subjects had been older women, who have more significant cardiovascular risk.

4 Triglycerides are the main component of body fat in humans. They do not pass readily through cell membranes, and they are major components of very-low-density lipoproteins (VLDLs), which are converted in the blood to LDLs. High levels of triglycerides in the blood have therefore been linked to atherosclerosis, heart disease, and stroke.

## Glossary

Actin: A family of globular multifunctional proteins, each of which forms (together with myosin) the contractile filaments of muscle cells, and is also involved in motion in other types of cells
Afterload: The pressure in the left ventricle wall during ejection of blood; the end load against which the heart contracts to eject blood
Alpha-Enolase: Also known as enolase 1 (ENO1). A glycolytic enzyme expressed in most tissues, one of the isoenzymes of enolase. Functions as a glycolytic enzyme and as a structural lens protein, with a shorter isoform functioning as a tumor marker
Alpha [α]-Myosin: A protein of the myofibril (elongated contractile thread in striated muscle cells); with actin, forms actomyosin, responsible for the contractile properties of muscle
Angiotensin I Receptor: A cellular receptor responsible for signal transduction of the vasoconstricting stimulus of angiotensin II, the main effector hormone; important in the *renin–angiotensin–aldosterone system (RAAS),* which regulates plasma sodium concentration and arterial blood pressure
Antioxidant Protein 2: Antioxidant that affects certain disease processes, including *atherosclerosis*
Apolipoprotein A-I: The major protein component of high-density lipoprotein (HDL) in plasma, or “good cholesterol”; plays a specific role in lipid metabolism
Atherosclerosis: A disease of the arteries characterized by deposition of plaques (made up of fats, cholesterol, cellular waste products, calcium, and fibrin) on arterial inner walls, which can restrict blood flow; commonly called hardening of the arteries
Atrogin: Protein expressed during muscle atrophy; atrogin-1 is expressed in cardiac and skeletal muscle and directs muscle protein degradation after muscle atrophy response has been triggered
Autophagy-Related 7 Protein: Myocardial marker of autophagy; also thought to modulate tumor-suppression protein–dependent cell cycle pathways during prolonged metabolic stress
Baroreflex: Also known as baroreceptor reflex; one of the body’s homeostatic mechanisms that helps to maintain blood pressure at nearly constant levels; part of a rapid negative-feedback loop that can begin to act in less than the duration of a cardiac cycle (fractions of a second), and thus a key factor in dealing with postural hypotension, the tendency for blood pressure to decrease on standing due to gravity
BAX Protein: Apoptosis-regulator protein that promotes apoptotic cell death by interacting with and increasing the opening of the mitochondrial voltage-dependent anion channel (VDAC), which leads to loss in membrane potential and release of cytochrome c and other proapoptotic factors from the mitochondria
BCL-2 Protein: Apoptosis-regulator protein that inhibits apoptotic cell death
Body Mass Index (BMI): Quantifies the amount of tissue mass (muscle, fat, and bone) in an individual based on mass (weight) and height, with categories labeled underweight, normal weight, overweight, or obese; commonly accepted BMI ranges are underweight: under 18.5, normal weight: 18.5 to 25, overweight: 25 to 30, obese: over 30
Carotid Intima-Medial Thickness (cIMT): Measurement of the thickness of the carotid arteries, which supply the brain, used to assess cardiovascular disease risk, since increased thickness is a marker of early stages of heart disease; a cIMT test is done with ultrasound, takes 10 minutes, is painless, and involves no radiation exposure
C-Reactive Protein: Acute-phase reactant protein in blood plasma, secreted by the liver, whose level increases in response to inflammation; can help predict risk of heart disease or stroke
C-terminal Proendothelin-1: Circulating inflammatory marker, elevated during heart failure and acute myocardial infarction
Cross-Bridge Cycling: Four-step cycle of muscle contraction in skeletal and cardiac muscle; named for myosin protein heads (cross-bridges) of thick filaments in a sarcomere, the functional unit of a myofibril or contractile protein, which bind to and move along actin in the sarcomere’s thin filament, an interaction that is the molecular basis for force generation and movement in muscle cells
Cytochrome P450 2E1: Membrane protein expressed in high levels in the liver, responsible for fatty acid oxidation and conversion of ethanol to acetaldehyde and to acetate in humans; also metabolizes foreign chemical substances in the body, including toxic environmental chemicals and carcinogens
Endogenous Nitric Oxide Synthase (eNOS): Protective enzyme that produces most of the *nitric oxide* in the vascular system and helps prevent platelet aggregation and adhesion
Fatty Acid Ethyl Ester (FAEE): Nonoxidative metabolite of ethanol, sometimes used as a biomarker of alcohol consumption; intoxicated humans have high levels of FAEE in blood, pancreas, liver, and hair
Fibronectin: Glycoprotein that in soluble plasma form is a major protein component of blood plasma and plays a major role in cell adhesion, growth, migration, and differentiation and is important in wound healing and formation of blood clots
Glutathione: Antioxidant capable of preventing damage to important cellular components caused by *reactive oxygen species* such as free radicals, peroxides, lipid peroxides, and heavy metals
Glutathione Transferase 5: Metabolic isoenzyme that helps in detoxification by catalyzing the binding of the reduced form of *glutathione* to certain foreign chemicals*,* which makes these molecules less toxic
Glycogen Phosphorylase: Glycolytic enzyme that catalyzes the rate-limiting step in the breakdown of glycogen, the main storage form of glucose in the body
Glycoproteins: Proteins important to various cell–cell interactions, including white blood cell recognition; found on surface membranes of platelets and integral to bleeding cessation, formation of blood clots, and normal platelet aggregation and adherence to the endothelium
Interleukin: *Glycoprotein* produced by white blood cells for regulating immune response, including three dozen currently identified types
Isocitrate Dehydrogenase: Enzyme that catalyzes the third step of the citric acid cycle while converting NAD^+^ to NADH in the mitochondria
Isoprostane: Inflammatory mediator that augments the perception of pain and is a biomarker of oxidative stress; elevated levels may contribute to increased risk of heart attack in people taking certain kinds of nonsteroidal anti-inflammatory drugs
Long-Chain Fatty Acids (LCFAs): Chief components of dietary fats; turned into triglycerides and taken up into cells, where they are metabolized by the mitochondria and yield large quantities of ATP; ingestion through certain foods promotes lipid accumulation and insulin resistance
Long-Chain-Specific Acyl-CoA Dehydrogenase: Mitochondrial enzyme that participates in fatty acid metabolism; mitochondrial mutations in it may be associated with some forms of dilated cardiomyopathy, which enlarges and weakens the left ventricle, making it harder for the heart to pump blood
Lysosome: Membrane-bound organelle in most animal cells that contains hydrolytic enzymes that can break down almost all biomolecules; involved in cellular secretion, plasma membrane repair, cell signaling, energy metabolism, and waste disposal
Mechanistic (or Mammalian) Target of Rapamycin (mTOR): Kinase that regulates cellular metabolism, growth, and proliferation; inhibits T-cell proliferation and proliferative responses induced by certain cytokines
Mendelian Randomization: A epidemiologic method of using measured variation in genes of known function to examine the causal effect of a modifiable exposure on disease in nonexperimental studies
NADH Dehydrogenase: Flavoprotein that reversibly oxidizes NADH to NAD^+^ in mitochondria, in the metabolic pathway that cells use to oxidize nutrients, thereby releasing energy to reform ATP
Nicotinamide Adenine Dinucleotide Phosphate-Oxidase (NADPH Oxidase): Membrane-bound enzyme complex found in plasma membrane that faces the extracellular space; major cause of *atherosclerosis* from its production of *reactive oxygen species* and resulting accumulation of cholesterol-containing macrophages in arterial walls
Nitric Oxide: Important cellular signaling molecule involved in many physiological and pathological processes; integral to vasodilation and increased blood flow in blood vessels; acts as powerful vasodilator with a short half-life of a few seconds in the blood; can contribute to reperfusion injury after ischemia
Pentraxin-3: Circulating inflammatory marker integral to protection against pathogens and to control of autoimmunity; facilitates pathogen recognition by macrophages and dendritic cells; appears to be primarily protective in both acute infections and acute coronary syndromes
Peroxiredoxin 5: Protein that acts protectively as an antioxidant in different tissues under normal conditions and during inflammatory processes; cardioprotective functions still to be determined
Phenylephrine: Selective α1-adrenergic receptor used as a decongestant and also as a vasopressor to increase blood pressure in patients with reduced blood pressure, especially from septic shock
Phosphatidylethanol: Phospholipid formed only in the presence of ethanol, used as a direct biomarker of previous alcohol consumption
Prostanoid: Signaling molecules that include prostaglandins, which mediate inflammatory and anaphylactic reactions; thromboxanes, which mediate vasoconstriction and help form blood clots (*thrombosis*); and prostacyclins, which help resolve inflammation
Reactive Oxygen Species (ROS): Chemically reactive molecules containing oxygen, formed as a natural byproduct of normal oxygen metabolism and integral to cell signaling and homeostatis; in times of environmental stress or with ionizing radiation, may result in significant damage to cell structures, known as oxidative stress
Renin–Angiotensin–Aldosterone System (RAAS): Neurohormonal system involved in regulation of plasma sodium concentration and arterial blood pressure; can be activated by loss of blood volume or drop in blood pressure; angiotensin-converting enzyme (ACE) inhibitors reduce formation of angiotensin II, a strong vasoconstrictor
Sarcoplasmic Reticulum: Smooth endoplasmic reticulum found in muscle cells that regulates the calcium ion concentration in the cytoplasm of striated muscle cells; stores calcium ions and pumps them into sarcoplasm when the muscle fiber is stimulated, thereby playing a major role in muscle contraction
Subarachnoid Hemorrhage: Bleeding into the subarachnoid space of the brain, the area between the arachnoid membrane and the pia mater, the brain’s innermost membrane; a form of stroke that can lead to severe disability or death
Thrombocytosis: Excessive production of platelets (thrombocytes) in the body; often without symptoms, but can cause *thrombosis* or formation of blood clots
